# Supplementary figures and images for: Growth faltering or deceleration toward target height: Linear growth interpretation using WHO growth standard 2006 for Indonesian children
Source: PLoS One. 2025 Apr 4;20(4):e0290053. doi: 10.1371/journal.pone.0290053 (PMC11970694; doi:10.1371/journal.pone.0290053)

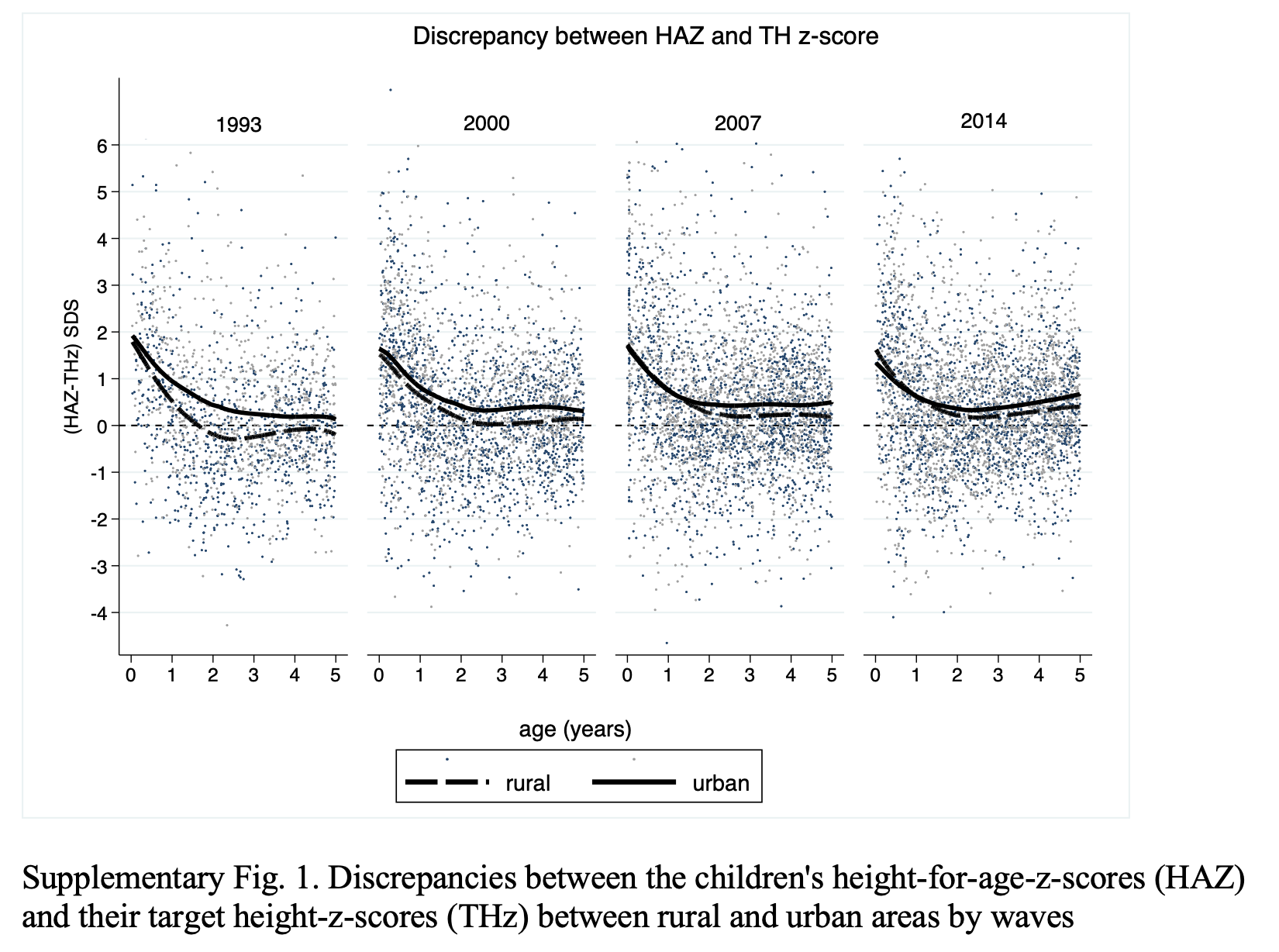

Supplement: S1 Fig — (TIF) [file pone.0290053.s001.tif]
