# Supplementary material for: Growth faltering or deceleration toward target height: Linear growth interpretation using WHO growth standard 2006 for Indonesian children
Source: PLoS One. 2025 Apr 4;20(4):e0290053. doi: 10.1371/journal.pone.0290053 (PMC11970694; doi:10.1371/journal.pone.0290053)
Supplement: S1 Table — (DOCX) [file pone.0290053.s002.docx]

**S1 Table 1. Discrepancies between the children's length or height-for-age z-scores (HAZ) and their target height z-scores (THz) in several age groups based on waves**

| **Age (months)** | **Difference between HAZ and THz (95% CI)** | | | | | | | | |  |
| --- | --- | --- | --- | --- | --- | --- | --- | --- | --- | --- |
|  | **wave 1993** | | **wave 2000** | | **wave 2007** | | **wave 2014** | | **p*** | |
|  | Δ | 95%CI | Δ | 95%CI | Δ | 95%CI | Δ | 95%CI |  | |
| **0–≤3** | 1.34 | (0.86-1.84) | 1.30 | (1.02-1.58) | 1.73 | (1.44-2.02) | 1.35 | (1.04-1.66) | 0.116 | |
| **>9–≤12** | 0.90 | (0.60-1.20) | 0.94 | (0.72-1.15) | 0.84 | (0.59-1.09) | 0.50 | (0.28-0.72) | 0.039 | |
| **>21–≤24** | -0.27 | ((-0.65)-0.10) | 0.07 | ((-0.16)-0.30) | 0.03 | ((-0.17)-0.24) | 0.17 | ((-0.03)-0.37) | 0.126 | |
| **>33–≤36** | 0.02 | ((-0.26)-0.31) | 0.17 | ((-0.07)-0.40) | 0.49 | (0.29-0.69) | 0.36 | (0.18-0.53) | 0.031 | |
| **>45–≤8** | 0.03 | ((-0.21)-0.28) | 0.38 | (0.18-0.58) | 0.33 | 0.15-0.52) | 0.33 | (0.14-0.52) | 0.162 | |
| **>57–<60** | (-0.10) | ((-0.36)-0.15) | 0.26 | (0.06-0.47) | 0.34 | (0.15-0.54) | 0.57 | (0.41-0.73) | <0.001 | |

*one-way ANOVA test
