# Supplementary material for: Growth faltering or deceleration toward target height: Linear growth interpretation using WHO growth standard 2006 for Indonesian children
Source: PLoS One. 2025 Apr 4;20(4):e0290053. doi: 10.1371/journal.pone.0290053 (PMC11970694; doi:10.1371/journal.pone.0290053)
Supplement: S2 Table — Univariate and multivariate analysis on predictors of height-for-age z-score (HAZ) in children aged 0- < 6 months, 6- < 24 months, and > 24 months. (DOCX) [file pone.0290053.s003.docx]

**S2 Table 2a. Univariate and multivariate analysis of height-for-age z-score (HAZ) in subjects aged 0-<6 months**

|  | **Univariate** | |  |  | **Multivariate** | |  |
| --- | --- | --- | --- | --- | --- | --- | --- |
|  | **Coeff.** | **95%CI** | **p** | **R2** | **Coeff.** | **95% CI** | **p** |
| THz | 0.37 | 0.21-0.54 | 0.000* | 0.018 | 0.37 | 0.21-0.53 | 0.000* |
| Male (female/reff) | (-0.23) | (-0.44)-(-0.02) | 0.034* | 0.003 | (-0.19) | (-0.39)-0.005 | 0.057 |
| BAZ | (-0.32) | (-0.38)-(-0.26) | 0.000* | 0.100 | (-0.34) | (-0.39)-(-0.28) | 0.000* |
| Number of household member | (-0.01) | (-0.04)-0.03 | 0.758 | 0.000 | 0.01 | (-0.02)-0.04 | 0.520 |
| Household expenditure share | (-0.58) | (-1.16)-0.03 | 0.051 | 0.003 | (-0.34) | (-0.93)-0.24 | 0.257 |
| Father’s education | 0.02 | (-0.001)-0.04 | 0.064 | 0.002 | 0.02 | (-0.02)-0.05 | 0.336 |
| Mother’s education | 0.02 | (-0.002)-0.04 | 0.075 | 0.002 | (-0.001) | (-0.04)-0.03 | 0.959 |
| Java-Bali islands (Outer/reff) | (-0.05) | (-0.16)-0.06 | 0.333 | 0.000 | (-0.05) | (-0.15)-0.06 | 0.313 |
| Urban (rural/reff) | 0.14 | (-0.07)-0.35 | 0.185 | 0.001 | (-0.01) | (-0.22)-0.20 | 0.892 |
| Wave (wave 1993/reff) |  |  |  |  |  |  |  |
| - wave 2000 | (-0.05) | (-0.42)-0.31 | 0.779 |  | (-0.10) | (-0.51)-0.30 | 0.602 |
| - wave 2007 | 0.06 | (-0.31)-0.42 | 0.761 |  | 0.18 | (-0.25)-0.61 | 0.415 |
| - wave 2014 | 0.04 | (-0.42)-0.33 | 0.817 | -0.002 | (-0.10) | (-0.54)-0.34 | 0.641 |
| Adj. R2 |  |  |  |  | 0.13 |  |  |

HAZ: height-for-age z-score; BAZ: body mass index-for-age z-score; *p<0.05

**S2 Table 2b. Univariate and multivariate analysis of height-for-age z-score (HAZ) in subjects aged 6-<24 months**

|  | **Univariate** | |  |  | **Multivariate** | |  |
| --- | --- | --- | --- | --- | --- | --- | --- |
|  | **Coeff.** | **95%CI** | **p** | **R2** | **Coeff.** | **95% CI** | **P** |
| THz | 0.54 | 0.46-0.62 | 0.000* | 0.050 | 0.52 | 0.44-0.59 | 0.000* |
| Male (female/reff) | (-0.16) | (-0.26)-(-0.05) | 0.003* | 0.002 | (-0.10) | (-0.20)-(-0.005) | 0.037* |
| BAZ | (-0.31) | (-0.34)-(-0.27) | 0.000* | 0.085 | (-0.33) | (-0.36)-(-0.29) | 0.000* |
| Number of household member | 0.0004 | (-0.02)-0.02 | 0.967 | 0.000 | 0.01 | (-0.004)-0.03 | 0.146 |
| Household expenditure share | (-0.73) | (-1.02)-(-0.45) | 0.000* | 0.007 | (-0.25) | (-0.53)-0.03 | 0.083 |
| Father’s education | 0.02 | 0.01-0.03 | 0.000* | 0.005 | 0.01 | (-0.02)-0.05 | 0.535 |
| Mother’s education | 0.02 | 0.01-0.03 | 0.000* | 0.005 | 0.003 | (-0.01)-0.02 | 0.710 |
| Java-Bali islands (Outer/reff) | (-0.19) | (-0.24)-(-0.13) | 0.000* | 0.014 | (-0.17) | (-0.22)-(-0.12) | 0.000* |
| Urban (rural/reff) | 0.34 | 0.24-0.44 | 0.000* | 0.012 | 0.14 | 0.04-0.24 | 0.007* |
| Wave (wave 1993/reff) |  |  |  |  |  |  |  |
| - wave 2000 | 0.08 | (-0.09)-0.25 | 0.362 |  | 0.06 | (-0.13)-0.24 | 0.504 |
| - wave 2007 | 0.14 | (-0.03)-0.30 | 0.108 |  | 0.11 | (-0.08)-0.30 | 0.238 |
| - wave 2014 | 0.09 | (-0.07)-0.25 | 0.279 | 0.000 | 0.01 | (-0.18)-0.20 | 0.875 |
| Adj. R2 |  |  |  |  | 0.16 |  |  |

HAZ: height-for-age z-score; BAZ: body mass index-for-age z-score; *p<0.05

**S2 Table 2c. Univariate and multivariate analysis of height-for-age z-score (HAZ) in subjects aged 2-<5 years**

|  | **Univariate** | |  |  | **Multivariate** | |  |
| --- | --- | --- | --- | --- | --- | --- | --- |
|  | **Coeff.** | **95%CI** | **p** | **R2** | **Coeff.** | **95% CI** | **p** |
| THz | 0.69 | 0.65-0.73 | 0.000* | 0.116 | 0.59 | 0.55-0.64 | 0.000* |
| Male (female/reff) | (-0.03) | (-0.09)-0.03 | 0.330 | 0.000 | 0.07 | 0.02-0.13 | 0.010* |
| BAZ | (-0.06) | (-0.08)-(-0.04) | 0.000* | 0.004 | (-0.08) | (-0.10)-(-0.06) | 0.000* |
| Number of household member | (-0.01) | (-0.02)-0.004 | 0.225 | 0.000 | 0.02 | 0.01-0.03 | 0.000* |
| Household expenditure share | (-0.94) | (-1.10)-(-0.77) | 0.000* | 0.018 | (-0.16) | (-0.33)-0.003 | 0.054 |
| Father’s education | 0.06 | 0.05-0.06 | 0.000* | 0.047 | 0.01 | 0.005-0.02 | 0.004* |
| Mother’s education | 0.06 | 0.05-0.06 | 0.000* | 0.050 | 0.02 | 0.01-0.03 | 0.000* |
| Java-Bali islands (Outer/reff) | (-0.13) | (-0.16)-(-0.10) | 0.000* | 0.011 | (-0.10) | (-0.13)-(-0.07) | 0.000* |
| Urban (rural/reff) | 0.47 | 0.41-0.53 | 0.000* | 0.034 | 0.24 | 0.18-0.30 | 0.000* |
| Wave (wave 1993/reff) |  |  |  |  |  |  |  |
| - wave 2000 | 0.22 | 0.12-0.32 | 0.000* |  | (-0.03) | (-0.14)-0.08 | 0.635 |
| - wave 2007 | 0.46 | 0.37-0.56 | 0.000* |  | 0.11 | (-0.001)-0.22 | 0.051 |
| - wave 2014 | 0.51 | 0.42-0.61 | 0.000* | 0.021 | 0.11 | (-0.003)-0.23 | 0.056 |
| Adj. R2 |  |  |  |  | 0.17 |  |  |

HAZ: height-for-age z-score; BAZ: body mass index-for-age z-score; *p<0.05
