# Supplementary material for: Growth faltering or deceleration toward target height: Linear growth interpretation using WHO growth standard 2006 for Indonesian children
Source: PLoS One. 2025 Apr 4;20(4):e0290053. doi: 10.1371/journal.pone.0290053 (PMC11970694; doi:10.1371/journal.pone.0290053)
Supplement: Attachment — Submitted filename: Response to reviewers.docx [file pone.0290053.s009.docx]

**Responses from reviewer #4**

***1. Relevance to Objectives***

*Recommendations:*

- *Ensure the conclusion is strong enough to take the message of how these findings are additive to the broader discourse on growth assessment with WHO Growth Standards.*
- *Discuss the potential public health implications of these findings more explicitly.*

**Response to 1:**

Thank you very much for the recommendations. We try to revise the manuscript as per your suggestions (yellow hightlights):

- We have added “*the WHO Growth Standards 2006*” in the conclusion, both in the abstract and the text (lines 40 and 293) and how the findings contributed to the growth assessment (in the conclusion of the text lines 296-299)

- We discuss *the potential public health implications of these findings more explicitly* (lines 252-263).

***3. Response to Reviewer Comments***

*Recommendations:*

- *While the responses to the reviewers are quite detailed, summarize explicitly how the additional analyses answer the larger aims of the study.*

Response: we summarize the additional analyses in a sentence (lines 270-272).

- *Format supplementary materials such as figures and tables according to journal guidelines.*

Response: We had formatted the figures in TIFF as journal guidelines mentioned.

- ***Critical Note:*** *Tables S1 and S2 are currently not accessible for review. The results derived from these tables must be explicitly referred to in the results section, and their main findings should be clearly discussed. Without this, it is challenging to assess their appropriateness and contribution to the manuscript's conclusions.*

**Response:** We have added a brief explanation of Tables S1 and S2 in the results (lines 192-194; 217-223) and in the discussion (lines 242-245; 239-241).

- *Reference Addition: For lines 59, 60, and 61 discussing critiques of the WHO Growth Standards, consider adding the following recent references to strengthen this section:*

1. *https://doi.org/10.1186/s12874-023-02116-y*
2. *https://doi.org/10.1111/apa.16770*

- *Similarly other references may be viewed and updated wherever possible (most of the references are old)*
- *For instances I can recommend for replacement of reference “1” which is from 2008 with*

1. *https://doi.org/10.1002/ajhb.23597*
2. *https://doi.org/10.1177/13674935211024816*

Response: We have added the references which were recommended and updated the references (ref: 1, 2, 4-6, 7, 8, 9, 10, 11, 12-15, 27, 30).

***4. Readiness for Acceptance***

*Suggestions for Final Review:*

- *Check if the discussion part is clear and readable, especially where new material has been incorporated.*
- *Supplementary materials should be of good quality and add to the value of the main manuscript.*
- *Check adherence to the formatting and submission guidelines of the journal, including legends for figures and captions for tables.*
- *The authors are commended for their efforts in addressing reviewer feedback and strengthening the manuscript. The study's findings meaningfully contribute to understanding child growth patterns and the need to consider parental height when interpreting growth deviations. This work has significant implications for growth assessment and public health policy in similar settings.*
- *Minor clarifications,* ***updating of references*** *and formatting checks will be enough to get the manuscript accepted.*

**Response to 4:**

Thank you for your review and suggestions.
We have reviewed the discussion section for clarity and readability.
We have also ensured that the supplementary materials add value to the main manuscript.
Additionally, we have checked the formatting, figures, and tables to ensure they adhere to the journal's guidelines.
Finally, we have verified the updated references.
