# Supplementary material for: Growth faltering or deceleration toward target height: Linear growth interpretation using WHO growth standard 2006 for Indonesian children
Source: PLoS One. 2025 Apr 4;20(4):e0290053. doi: 10.1371/journal.pone.0290053 (PMC11970694; doi:10.1371/journal.pone.0290053)
Supplement: Attachment — Submitted filename: Responses to reviewers.docx [file pone.0290053.s005.docx]

# **Responses to reviewers**

| **No** | **Note** | **Responses** |
| --- | --- | --- |
| **Reviewer 1** | |  |
| Major |  |  |
| 1 | **Introduction**  • Based on the text from the introduction and findings from Roth et al, 2017 study that authors quote at stating that community-level factors might be behind the downward shifts of HAZ distribution in the entire population. It is not clear for me, why authors hypothesized that genetic target height, an individual-level factor, might be the factor behind the shifts of the HAZ distribution among Indonesian children. | Thank you very much for your recommendation, we realized that the word 'genetic' could be misleading.  We replaced the term “genetic target height” into “target height” to avoid confusion because of the word “genetic”. The explanation on the definition of “target height” had been added in the Introduction. (line 65-72)  Although target height is calculated individually for every parents-child pair, the same formula is used for every parents-child pair in the population. (line 139-144) Our study wanted to test whether these individual target heights directed the shift of HAZ of every child in the population. Of course, parental heights did exhibit secular trends as shown by the increase of the parental height means from wave 1 to wave 4 (table 2). |
| 2 | • Page 3: “The classic concept of growth acceleration or deceleration toward their genetic target height in the first few years of life might become a plausible explanation for this 'universal' growth faltering.” Could authors talk a little bit more on the genetic target height, what does it mean? Are there previous studies in Indonesia or another LMICs that authors can talk about? | Thank you very much for the recommendation, we decided to re-write this part for better clarity.  The explanation on the definition of “target height” is already added in the Introduction and Methods. (line 65-67; line 139-144)  As far as we know, there has been no recent studies that hypothesize the possibility of deceleration toward target height as a possible explanation to what seemed to be 'growth faltering' of every child in the population. This is the novel part of our study. |
| 3 | • Page 3: “Our previous observation showed that Indonesian adults were relatively short compared to WHO 2007 reference.12”. Is this mismatch purely genetic? Or is it the result of generational undernutrition?  Studies using archeological evidence like bones of ancient individuals living in India (11,000 years before the present and in the Mesolithic time),1,2 showed that these communities were taller than Europeans, and there has been a reduction in height through the centuries as a result of climatic adaptations, the transition to agriculture, and exposure to nutritional deficits specially during the British colonization (19th and 20th century), factors that might have contributed to the current growth faltering in India.  I also understand from your previous findings on adult height in Indonesia3 that proxies of socioeconomic factors such educational attainment and share of household expenditure are associated positively with a higher height in adult life. Thus, this demonstrates that beyond genetic characteristics, there are macrolevel factors that might be associated with growth faltering in Indonesia.  Evidence from GWS (genome-wide association studies) have also shown that between 40-50% of height is genetically determined, especially among people from European ancestry;4,5 it seems that among people from other ancestries could be a lower percentage, but more information is needed. This suggests that there remaining percentage might be determined by nutrition and other environmental factors, that have key role at influencing growth and the final attained size since early life and across the life course.  Therefore, based on these findings and acknowledging that the WHO growth standards showed solid evidence on the potential to grow of all humans, independent of their genetic and ethnic background, if children have adequate conditions (food, support, are free of disease) to grow and develop, they will have a similar potential to attain the same size as they grow older, especially during the first 5 years of life.  In summary, I wonder what the rationale and evidence is to support the hypothesis that growth deceleration towards the genetic target height is the reason behind the downward shift in HAZ distribution instead of growth faltering across the entire population of children younger than 5 years of age. It is not clear from the evidence presented what would be the limitations of the WHO growth standards, and why the shifts in the distribution of HAZ among Indonesian children are not a reflection of growth faltering across the entire population (a common phenomenon in low-and middle-income countries, as Roth et al 2017 showed in an analysis of data from 64 LMICs).  References  1.Lukacs, J. R., & Pal, J. N. Skeletal Variation among Mesolithic People of the Ganga Plains: New Evidence of Habitual Activity and Adaptation to Climate. Asian Perspectives. 2003 42(2), 329–351.  2.Pomeroy E, Mushrif-Tripathy V, Cole TJ, Wells JCK, Stock JT. Ancient origins of low lean mass among South Asians and implications for modern type 2 diabetes susceptibility. Sci Rep. 2019 Jul 19;9(1):10515.  3.Moelyo AG, Sitaresmi MN, Julia M. Secular trends in Javanese adult height: the roles of environment and educational attainment. BMC Public Health. 2022 Apr 11;22(1):712.  4.Yengo, L., Vedantam, S., Marouli, E. et al. A saturated map of common genetic variants associated with human height. Nature 610, 704–712 (2022).  5.Yang J, Benyamin B, McEvoy BP, Gordon S, Henders AK, Nyholt DR, Madden PA, Heath AC, Martin NG, Montgomery GW, Goddard ME, Visscher PM. Common SNPs explain a large proportion of the heritability for human height. Nat Genet. 2010 Jul;42(7):565-9. | Thank you very much for the suggestions. Indonesians are indeed shorter than the Americans whose data were used to construct the WHO 2007 growth reference. However, to assume that the whole nation underwent undernutrition just because its population is shorter is too presumptuous. The GDP per capita of Indonesia was USD 824 in 1993, which increased more than four times to USD 3480 in 2014 without a significant increase in both adult heights and children's heights. (<https://datacommon.org>)  We agree that colonization may have impacts on human heights. Indonesians were much shorter during the Dutch colonization (van der Eng et al., 2010)  Yes, you are right. Our previous study indeed showed the association between educational attainment and household expenditure shares with adult heights (Moelyo A et al., 2022). However, this study showed that the growth of children whose parents were at the upper part of the height distribution exhibited a similar phenomenon to children whose parents were at the lower part. All population members grew toward their target height (Figure 2).  You are absolutely right. Our findings supported the theory that around 40-50% of height was genetically determined, as shown in supplementary table 2 a, b, and c. The older the child, the larger is the association between the child's height and his/ her parents' height.  Concerning WHO Growth Standard 2006, we beg to disagree. The presupposition that all children should grow according to the WHO Growth Standard 2006 has long been questioned. Many European countries have stopped using the WHO Growth Standard 2006 because their children have grown taller than the WHO growth standard 2006 (Christesen et al. 2016; Bonthuis et al., 2012), not to mention many developed countries that have never used the WHO Growth Standard 2006 in the first place, such as Japan and the United States.  References:   - Christesen HT, Pedersen BT, Pournara E, Petit IO, Júlíusson PB (2016) Short Stature: Comparison of WHO and National Growth Standards/ References for Height. PLoS ONE 11(6): e0157277. doi:10.1371/journal.pone.0157277. - Bonthuis M, van Stralen KJ, Verrina E, Edefonti A, Molchanova EA, Hokken-Koelega ACS, et al. (2012) Use of National and International Growth Charts for Studying Height in European Children: Development of Up-To-Date European Height-For-Age Charts. PLoS ONE 7(8): e42506. https://doi.org/10.1371/journal.pone.0042506 - van der Eng P, Baten J, Stegl M. Long-term economic growth and the standard of living in. Indonesia; 2010. https:// doi. org/ 10. 2139/ ssrn. 1699972. |
| 4 | **Methods**  • The manuscript is missing some important details in the method sections based on STROBE guidelines for cross-sectional studies. Please provide more details about the data collection and for each variable of interest, give sources of data and details of methods of assessment (measurement). How did they get information about parents? Was the anthropometric data measured or self-reported in children and parents? Please provide information about the selection of the analytic sample. | We have added some information to fulfill STROBE guidelines. (line 82-132) |
| 5 | • Statistical analyses can be improved. Authors only did a characterization of analytic sample and stratified by sociodemographic characteristics and showed differences in targeted height by these characteristics. I wonder why authors did not run regression analyses to test for differences among groups but at the same time to control for potential confounders (nutrition deficiencies, socioeconomic status, illness). Did the analyses include survey weights and other variables (cluster and stratum) that consider the survey design? (Important aspects to be able to make generalizations to the population level) | Thank you for the recommendation. This paper was not, in the first place, intended to look for individual or household-level risk factors. However, as supplementary, we have added supplementary Table 2 a, b, and c to show that, after controlling for potential confounders, e.g, sex, BAZ, number of household members, household expenditure share, parental education, urban/ rural, etc., THz remained the most important determinant of the children's HAZ. (line 207-211) |
| 6 | • I wonder what the proportion of stunting in your study population is? It would be interesting to do stratify analysis by children with normal length at birth vs children with stunting at birth, and/or to control for birth weight and birth length. | Thank you for your recommendation.  WHO's definition of stunting is ' Stunting is the impaired growth and development that children experience from poor nutrition, repeated infection, and inadequate psychosocial stimulation. Children are defined as stunted if their height-for-age is more than two standard deviations below the WHO Child Growth Standards median.' (Stunting in a nutshell. https://www.who.int/news/item/19-11-2015-stunting-in-a-nutshell)  Since our data can only show the number of children whose length or height was 2SD below the WHO 2006 Growth Standard median and cannot show which child had poor nutrition, repeated infection, or inadequate physical stimulation, we decided not to report stunting as the results because it will violate the WHO definition of stunting.  We cannot stratify children into normal birth length vs. children with stunting at birth because this was a multiple cross-sectional survey. |
| 7 | **Discussion**  • Not sure if authors can make conclusions that the differences in height-for-age z-scores between children and their parents (genetic targets) are a natural adaptation to genetic height.  In this analysis, authors did not control for socioeconomic status, and other potential confounders. With the information provided, I am not sure if the study population had children with good health and nutritional status. I also think, if children had stunting at birth, there is a possibility that they might growth faster in the first two years as a reflection of catch-up growth and then reach their expected trajectory as they grow older. | Thank you very much for the recommendation.  Although it was not our objective to look for individual or household level risk factors, we add supplementary Table 2 a, b, c to show that after controlling for potential confounders, e.g., sex, BAZ, number of household members, household expenditure share, parental education, urban/ rural, etc., THz remained the most important determinant of the children's HAZ. (line 207-211)  Your second sentence assuming that none of the study population were children with good health and nutritional status was like assuming all Indonesians were of poor nutrition and poor health because subjects were randomly selected from the population. Are you sure that is what you are intending to say? Furthermore, Table 1 showed that approximately 85% of the population had normal weight, 3 to 7% was overweight/ obese. (line 169)  Your third sentence is absolutely correct. As we have stated in the Introduction, children may catch up (accelerate against the growth chart) or catch down (decelerate against the growth chart) according to his/ her present height position in relation to his/ her target height. However, our findings showed that most Indonesians were born longer than their target heights because the dominant pattern was the deceleration of growth. |
| 8 | • Page 7: “When the parents' height increases, so will the child's target height and HAZ.” Please add a reference to support this statement. In contrast to this statement, other authors have also shown that children with parents of short height will have the potential to attain a taller size. There is a secular trend of increments in height in the recent generations at the population level, especially in LMICs. (Cole TJ. The secular trend in human physical growth: a biological view. Econ Hum Biol. 2003 Jun;1(2):161-8) | Thank you very much, we completely agree. We add the paper by Cole, 2003 in our references. What we observed was completely in agreement with what was reported by Cole in 2003. (line 225-228)  We indeed observed that children of parents of short height had the potential to attain a taller size, as shown by larger discrepancies between HAZ-THz in children of short parents compared to children of tall parents (Figure 2) (line 188-197)  We have revised the sentence: “When the parent’s height increases, so will the child’s target height.” (Table 2) (line 229-234) |
| 9 | Page 8: Our study found an interesting finding in overweight–obese children. They decelerated  earlier to rebound earlier and higher compared to the other nutritional status group. Due to the cross-sectional nature of our data, it was difficult to explain this phenomenon. This phenomenon has already been described in other populations using birth cohorts. Additionally, analyses on children with obesity seem disconnected from the study aim and research question. It should be tightly connected throughout the paper and included in the introduction. | Thank you very much, you are absolutely right, the discussion about overweight-obese children seemed to be out of the aim and the research questions. We delete this finding and all discussions related to overweight-obese children. |
| 10 | The potential limitations of this analysis are missing in the discussion section. | We add a paragraph considering potential limitations. (line 253-259) |
| **Minor** |  |  |
| 1 | **Introduction** – Page 2: “The causes of the observed low mean length or height z-scores (HAZ) and the high prevalence of stunting in those countries have been subjected to many epidemiological studies, primarily focusing on individual or household-level risk factors.2”  Please be careful, this sentence seems like a replica of the original article (reference 3) | Thank you very much for the reminder. We should have been aware of the similarity. We have changed the sentence and added another reference. (line 47-48) |
| **Reviewer 2** | |  |
| 1 | Introduction - generally clear although lacking current background data on the burden of the issue and related health consequences in your population. Would help if current prevalence of 'stunting'/growth faltering is provided as reference # 4 is based IFLS data upto 2007, rather outdated data to quantify population burden in present day Indonesia | Thank you very much for the recommendation.  We add references with updated data on the prevalence of 'stunting' in Indonesian children under five years of age. (line 56-59) |
| 2 | **Methods**  Lacking some description on anthropometric measurements and their quality, for example who measured height data? were they trained on standardized protocols? What was done to ensure reliable measurements over the 4 cycles of data used in this study? Such information could help address quality assurance issues. | Thank you very much for the recommendation.  We have added details on anthropometric measurements in methods based on the information available in the IFLS. (line 119-132) |
| 3. | Construct of target height. Not sure the use of THZ is clear as discerned here as data is serial cross-sectional data of different children. Parental height is their adult height, their own height during childhood is not available in this dataset so how are authors connecting child HAZ to parental THZ here? Kindly clarify. | Thank you very much for the recommendation.  A detailed explanation of the definition of target height and target height z-score has been added in the Introduction. This study used cross-sectional data of parent-child pairs. We relate the child's current height to his/ her target height, calculated from parental heights. Our hypothesize is that, as a child grows into his or her target height, the older the child, the closer his or her HAZ to his or her target height z-score (THz) (line 65-72) |
| 4 | Also genetic potential of growing children is not achieved until adulthood, so quantifying/characterizing SD score changes as deceleration toward genetic target height without longitudinal data challenging as positive or negative differences in HAZ-THZ are meaningful as they are SD scores at different ages. Could this be further clarified? | You are right. The most appropriate method to address this issue is using longitudinal observations. We have added this as our limitations. (line 253-259)  However, as we have thousands cross-sectional observations of parent-child pairs, we can examine the HAZ trend from different ages of children against their THz |
| 5 | **Discussion:**  Generally clear but as discerned from text, struggling to capture the fundamental construct of extrapolating parental height to diagnose linear growth faltering of their offspring during childhood. Would help if this is carefully discussed because growth faltering or 'stunting' is a proxy of many other factors (maternal status, nutrition, intra-uterine growth, intergenerational transfer of environmental insults etc. etc. ) with the role of genetics being weaker in early life and picking up more during adolescence through early adulthood. | Thank you very much for the recommendation  Although it was not our objective to look for individual or household level risk factors, we add supplementary Table 2 a, b, c to show that after controlling for potential confounders, e.g., sex, BAZ, number of household members, household expenditure share, parental education, urban/ rural, etc., THz remained the most important determinant of the children's HAZ. (line 174)  The table also shows that the contribution of THz to HAZ is larger as the children get older. It confirmed previous findings that the role of genetics is weaker in early life and becomes stronger after that. In our study, the target height was weaker in subjects under six months of age and stronger after that. (line 174) |
