# Supplementary material for: Growth faltering or deceleration toward target height: Linear growth interpretation using WHO growth standard 2006 for Indonesian children
Source: PLoS One. 2025 Apr 4;20(4):e0290053. doi: 10.1371/journal.pone.0290053 (PMC11970694; doi:10.1371/journal.pone.0290053)
Supplement: Attachment — Submitted filename: Responses_to_reviewers_auresp_3.docx [file pone.0290053.s008.docx]

**Responses to reviewer #3**

1. *They state the height for age z-scores of the child tends to approach the target height irrespective of the birth z score being above or below what is predicted.*

*The authors claim is that there is a difference between “faltering of growth” due to environmental factors and “deceleration toward target height” due to a physiological set point for height that is determined by the parents heights. And they further claim that short stature in Indonesia is due to short parents, and not necessarily conditions that have an adverse effect on child growth. Their main evidence of this is that children tend to be born at a length greater than expected, and across multiple socio-economic groups, although the latter is not actually shown in the data.*

**Response to #1:**

Thank you very much for the recommendations. We will try to response to your inquiries and, if applicable, to revise the manuscript accordingly.

Previous study by Dwipoerwantoro PG et al (2015) (reference no. 9 in our manuscript), had criticized that the WHO growth standards did not reflect the growth of the present cohort of Indonesian infants and may overestimate the levels of underweight and stunted children. Based on this observation, we tried to find out whether target height was the factor we should consider. Previous study by Leroy et al (2014) also mentioned that height deficit occurred in all regions, with different size of the deficit.

Our study was mainly inspired by the paper of Roth et al., in 2017. In the paper, Roth et al showed that the downward shift of growth trajectory observed when the growth of children in the developing countries were compared to the WHO 2006 growth standard, affected both the upper and the lower end of the height distributions. As they grew, the taller children ‘lost’ height standard deviation scores as much as those of the shorter children.

Due to this parallel downward shift, Roth et al also suggested to, instead of looking at the individual or household level determinants of growth, to look for community level determinants that influence both the taller and the shorter children simultaneously.

To show our claim that this downward shift in height-for-age z-scores affected the entire spectrum of the HAZ distribution, i.e across multiple socioeconomic status groups, we will add the following figures as supplementary figures, completing the existing figures.

Supplementary Figure 2. Height-for-age z scores stratified by waves of the data collection (year 1993, 2000, 2007 and 2014). Similar patterns were observed across waves.

Supplementary Figure 3. Height-for-age z scores stratified by quartiles of the household expenditure shares. Quartile 1 were children from the poorest households while quartile 4 were children from the richest households. Similar patterns, albeit of different magnitudes, were observed across all socioeconomic status groups, at least until the age of two years.

Supplementary Figure 4a and 4 b. Height-for-age z scores stratified by paternal and maternal highest educational achievement. Similar downward patterns were observed between children of highly educated parents and children of lowly educated parents, at least until the age of 2 years.

Supplementary Figure 5. Height-for-age z scores stratified by urban or rural areas of residence. Similar downward patterns were also observed between children who lived in the rural area and those who lived in the urban.

Supplementary Figure 6. Height-for-age z scores stratified by whether the children lived in Java/ Bali islands or outer Java/Bali. Islands of Java and Bali were considered more developed than those of outer Java/ Bali. Similar downward patterns were also observed.

The existing figures (Fig. 1 and Fig 2, as well as supplementary Fig. 1) have also shown that the discrepancies between target heights and the children’s current HAZ were consistent across waves and urban/ rural residences.

Figure 2, interestingly, showed that the lower the target heights, the bigger was the positive discrepancy between HAZ and THz, meaning children of relatively shorter parents had the potential to be taller than their parents, but not children of relatively taller parents.

We can add figures on the discrepancies between THz and HAZ stratified by quartiles of the household expenditure shares, parental educational achievements, and living in Java/Bali or not if it is deemed necessary.

Furthermore, Supplementary table 2a, 2b, 2c showed that target height had the largest coefficient compared to the other predictors of height, i.e household expenditure share, parental education, lived in Java-Bali islands or outer, and lived in urban/rural areas.

2. *But Figure 1 shows that children are typically born at a z-score greater than predicted by the parents height, and they then approach the predicted z-score as they get older; but for the 4 cohorts from 1993 to 2014, the height of the children progressively exceeds the predicted height. These data suggest that intrauterine growth is better than predicted based on maternal and paternal height, and would indicate that the conditions in pregnancy have improved since the time the parents were children, as would be expected. And that postnatal conditions have also improved, but not as much, as evidenced by the children exceeding the target height in the more recent cohorts.*

**Response to #2:**

We agree with the reviewers’ comments. Indeed, the height of the children progressively exceeds the predicted height. This observation is in concordance with our previous paper on the secular trends in heights of adults in Indonesia (Moelyo et al., 2022, reference no. 25). Indonesian’s final height increased approximately 1 cm every generation (men and women: 1.3 cm and 0.9 cm per decade) (in approximately 20 years).

We have added this information into the discussion section (line 226) (highlighted).

3. *Figure 2 shows clearly that the more the children exceed the target height at birth, the taller they tend to stay. The fact that all the children decline in height indicates that for most children the post-natal growth conditions are suboptimal, not that there is a target height. While it is true that the degree of decline tends to be similar for the various trajectories that start from birth at higher than predicted z-scores, this does not mean there is not growth faltering, but rather indicates there is growth both growth faltering and an impact of parental height on child height, the latter of which is well known for 40 years or more.*

**Response to #3:**

We agree with the reviewer’s comments. Interestingly, Supplementary table 2a, 2b, 2c showed that target height had the largest coefficient compared to the other predictors of height, i.e household expenditure share, parental education, lived in Java-Bali islands or outer, and lived in urban/rural areas.

It seemed that parental heights were better predictors of the children’s current heights compared to the other predictors observed in this study. Parental heights should be considered before labelling growth faltering or stunting on an individual child.

4. *To indicate there is a target height, it would also be useful to examine the trajectories of children born below the target height and to see if they have catch-up growth to the target height, especially after adjustment for socioeconomic status.*

**Response to #4:**

Thank you for the recommendation. It became one of the limitations of our study. Our data is cross sectional repeated surveys with 7 years gap between the surveys. Hence, we are sorry that we cannot show the growth trajectory of any individual child. We have stated this as the limitations of our study in line 272-278.

5. *The authors should also show the trajectories of each child cohort based on the actual child z-scores and not only the deviations from the predicted z-scores. And they should show the trajectories stratified by socioeconomic status, by deciles.*

**Response to #5:**

We are sorry that we cannot show the growth trajectory of any individual child because of the nature of our data. Supplementary figures 2,3,4,5 and 6 showed that better socioeconomic status showed better heights, however the deceleration patterns of height z-scores were similar.

6. *They should also show the scatterplot and regression analysis, and variance explained, in child height based on parental heights.*

**Response to #6:**

We provided the scatterplot below (Must we put this also in supplementary figure?). We showed the multivariate analysis in supplementary tables 2 a, b and c and showed that target height z-scores had the largest coefficient regression compared to the others.

Fig. Scatterplot and Regression line between HAZ and THz

7. *Overall, the data are most parsimoniously explained by points #3 and #4, and not by birth length being programmed to descend to the target height determined by the parents heights, and that therefore growth faltering is absent. The authors should also be mindful that the stunting of any specific child is difficult to state based on a single measurement, and that growth trajectories of each child are the most useful. As such, stunting in a population is the important indicator, as a healthy population would be expected to have approximately 2.5% of the children below -2 SD (De Onis et al., 2019).*

**Response to #7:**

We completely agree that stunting of any specific child is difficult to state based on a single measurement, and that growth trajectory of each child are the most useful. However, this study suggested that considering parental heights when diagnosing stunting in an individual child, was very important.

We also completely agree that a healthy population is expected to have approximately 2.5% of the children below -2 SD. However, as has been questioned by many countries who do not use the WHO Growth Standard 2006 to assess their children’s growths, whether the use of only the WHO Growth Standard 2006 to judge the growth of Indonesian children is appropriate. This is because using only the WHO Growth Standard 2006 to judge the growth of an individual child will ignore the influence of parental heights.

In this study we only reported the heights of Indonesian children in relation to their parents’ heights, and we clearly showed that parental heights influenced the children heights across times and socioeconomic status.

8. *The authors should review the literature on growth faltering and the basis for the changes in height seen in multiple populations that experience economic development. Data from Indonesia show that children from high socioeconomic households are indeed reaching increased heights with progressive increases in z-scores, indicating that growth faltering in Indonesia is decreasing, as would be expected given its economic development. As such, “deceleration toward target height” which has been previously proposed by many persons is lacking in evidence when it has been examined in detail, and it remains a fallacy based on known trends in height of children and of populations, and especially for children from impoverished situations who were later raised under proper conditions wherein they routinely exceed growth expectations. While there is clearly a hereditary component of height, factors other than that account for a substantial proportion of stunting in populations with high prevalence stunted children.*

**Response to #8:**

Thank you for the explanation from the reviewer. Previous study by Roth et al (2017) (#reference 3) has reported the growth trajectories of children in low-income and middle-income countries. It concluded that the dominant underlying causes of postnatal linear growth faltering are population-wide exposures (ie, community-level or ubiquitous factors to which nearly all children in the population are exposed) rather than distinct behaviors or characteristics of certain individuals or households. This manuscript clearly showed that parental heights influenced the heights of the children, across times and socioeconomic status. Although, off course, other factors also had roles.

Our previous study showed the secular trend of Indonesian adult height. The birth year, educational attainment, and share of household food expenditure significantly influenced adult heights (Moelyo et al., 2022).

9. *The authors should do the analysis that has been suggested. The authors can utilize the IFLS data, that is a longitudinal survey with various social, economic, health, and child growth data.*

**Response to #9:**

Thank you for the recommendations. IFLS surveys were done every 7 years, so, although it is possible to use longitudinal information, the long gap made it impossible to assess only childhood growth. It would be impossible to find the trajectories in each year of age or each 6 months of age.
