# Supplementary material for: Growth faltering or deceleration toward target height: Linear growth interpretation using WHO growth standard 2006 for Indonesian children
Source: PLoS One. 2025 Apr 4;20(4):e0290053. doi: 10.1371/journal.pone.0290053 (PMC11970694; doi:10.1371/journal.pone.0290053)
Supplement: Attachment — Submitted filename: Comments to authors.docx [file pone.0290053.s007.docx]

**General Evaluation**

In the present version of the manuscript entitled "Growth faltering or deceleration toward target height: Linear growth interpretation using WHO Growth Standard 2006 for Indonesian children", authors have done the substantial improvements compared to previous rounds of review; they addressed almost all concerns expressed previously, and now the manuscript matches its proposed goal well. Further below is the detailed commenting on the crucial parts of the manuscript, as well as on additional improvement possibilities where appropriate.

**1. Relevance to Objectives**

It is clear that the manuscript aims to assess whether the decline in HAZ in Indonesian children is a consequence of "growth faltering" or "deceleration toward target height." The analyses conducted, such as the comparisons between HAZ and THz, effectively support the stated objective. The argument is strengthened further with the addition of stratified analyses, for instance, by socioeconomic status, parental education, and geographic location.

**Recommendations:**

- Ensure the conclusion is strong enough to take the message of how these findings are additive to the broader discourse on growth assessment with WHO Growth Standards.
- Discuss the potential public health implications of these findings more explicitly.

2. Relevance and Appropriateness of Reviewers Comments

The reviewers' previous comments appropriately focused on:

- The distinction between "growth faltering" and "deceleration toward target height."
- Strengthening the evidence through stratified analyses and additional figures.
- Acknowledging data limitations, for instance, cross-sectional design, when making claims.
- Commenting on how socioeconomic and community-level factors may affect growth trajectories.
- The comments were very relevant and improved the quality of the manuscript by promoting rich evidence and filling potential holes in the argument.

3. Response to Reviewer Comments

- The authors have done a commendable job responding to the reviewers' comments:
- Data Stratification: Additional figures are now available with stratified analyses by socioeconomic factors, education of parents, and geographic locations. All of these analyses are showing consistent patterns across the different subgroups. This makes the conclusions drawn from the manuscript much stronger.
- Addressing Data Limitations: The authors have admitted that the data is cross-sectional and does not follow individual growth trajectories. The limitation has been stated clearly and discussed in the limitations section.
- Incorporation of Secular Trends: The authors have taken up the secular rise in height in Indonesia and incorporated it along with the cause that associates it with improvements in intrauterine and postnatal conditions.
- Scatterplots and Regression Analyses: The addition of scatterplots and regression analyses showing the relationship between parental height and child HAZ, is a good addition that reiterates the dominance of genetics in determining growth patterns.

**Recommendations:**

- While the responses to the reviewers are quite detailed, summarize explicitly how the additional analyses answer the larger aims of the study.
- Format supplementary materials such as figures and tables according to journal guidelines.
- **Critical Note:** Tables S1 and S2 are currently not accessible for review. The results derived from these tables must be explicitly referred to in the results section, and their main findings should be clearly discussed. Without this, it is challenging to assess their appropriateness and contribution to the manuscript's conclusions.
- Reference Addition: For lines 59, 60, and 61 discussing critiques of the WHO Growth Standards, consider adding the following recent references to strengthen this section:

1. https://doi.org/10.1186/s12874-023-02116-y
2. https://doi.org/10.1111/apa.16770

- **Similarly other references may be viewed and updated wherever possible (most of the references are old)**
- **For instance I can recommend for replacement of reference “1” which is from 2008 with**

1. https://doi.org/10.1002/ajhb.23597
2. https://doi.org/10.1177/13674935211024816

**4. Readiness for Acceptance**

The manuscript seems well and ready for acceptance based on the following strengths:

- The methodology is strong and aligns very well with the objectives.
- Reviewer concerns have been addressed substantially and transparently.
- Findings are well-supported through robust analyses and are presented very clearly.

Suggestions for Final Review:

- Check if the discussion part is clear and readable, especially where new material has been incorporated.
- Supplementary materials should be of good quality and add to the value of the main manuscript.
- Check adherence to the formatting and submission guidelines of the journal, including legends for figures and captions for tables.
- The authors are commended for their efforts in addressing reviewer feedback and strengthening the manuscript. The study's findings meaningfully contribute to understanding child growth patterns and the need to consider parental height when interpreting growth deviations. This work has significant implications for growth assessment and public health policy in similar settings.
- Minor clarifications, **updating of references** and formatting checks will be enough to get the manuscript accepted.
